# A new adult appendicitis score improves diagnostic accuracy of acute appendicitis - a prospective study

**DOI:** 10.1186/1471-230X-14-114

**Published:** 2014-06-26

**Authors:** Henna E Sammalkorpi, Panu Mentula, Ari Leppäniemi

**Affiliations:** 1 Department of Abdominal Surgery, Helsinki University Central Hospital, PL 340, 00029 HUS, Helsinkii, Finland

**Keywords:** Appendicitis, Sensitivity and specificity, Abdominal pain, Abdomen Acute, Abdomen Acute/etiology, Appendicitis/diagnosis, Blood cell count, C-reactive protein/analysis, Appendicitis score, Diagnostic score, Adults

## Abstract

**Background:**

The aim of the study was to construct a new scoring system for more accurate diagnostics of acute appendicitis. Applying the new score into clinical practice could reduce the need of potentially harmful diagnostic imaging.

**Methods:**

This prospective study enrolled 829 adults presenting with clinical suspicion of appendicitis, including 392 (47%) patients with appendicitis. The collected data included clinical findings and symptoms together with laboratory tests (white cell count, neutrophil count and C-reactive protein), and the timing of the onset of symptoms. The score was constructed by logistic regression analysis using multiple imputations for missing values. Performance of the constructed score in patients with complete data (n = 725) was compared with Alvarado score and Appendicitis inflammatory response score.

**Results:**

343 (47%) of patients with complete data had appendicitis. 199 (58%) patients with appendicitis had score value at least 16 and were classified as high probability group with 93% specificity.Patients with score below 11 were classified as low probability of appendicitis. Only 4% of patients with appendicitis had a score below 11, and none of them had complicated appendicitis. In contrast, 207 (54%) of non-appendicitis patients had score below 11. There were no cases with complicated appendicitis in the low probability group. The area under ROC curve was significantly larger with the new score 0.882 (95% CI 0.858 – 0.906) compared with AUC of Alvarado score 0.790 (0.758 – 0.823) and Appendicitis inflammatory response score 0.810 (0.779 – 0.840).

**Conclusions:**

The new diagnostic score is fast and accurate in categorizing patients with suspected appendicitis, and roughly halves the need of diagnostic imaging.

## Background

Acute appendicitis is the most common indication for emergency surgery worldwide, with incidence of 1.17 per 1000 and lifetime risk of 8.6% in men and 6.7% in women. The incidence is highest in adolescents and young adults, but the incidence of complicated appendicitis shows little variance between different age groups [1,2].

Although a very common and long-known phenomenon, appendicitis remains a diagnostic challenge for surgeons and emergency physicians. Clinical diagnosis alone leads to a negative appendectomy rate of 15 to 30%. The diagnosis is specially challenging for women of fertile age [3-5].

Early surgical intervention is the traditional gold standard for preventing appendicular perforation. High rate of unnecessary negative appendectomies, however, leads to unnecessary morbidity and even mortality [6,7].

The frequent use of computed tomography (CT) with its high sensitivity and specificity in diagnosis of appendicitis has helped to reduce the number of negative appendectomies [4,8,9]. Preoperative CT seems to benefit most women 45 years of age and younger [10,11]. The use of CT may, however, delay appendectomy in clinically typical cases of acute appendicitis, and therefore even elevate the risk for perforation [12,13]. Increased use of CT is associated with elevated risk of cancer especially in young patients, whose incidence of acute appendicitis is greatest [14].

Several scoring systems for diagnosing appendicitis already exist [15-21]. The best known is the Alvarado score.

An ideal scoring system would work as a tool that speeds up and increases the accuracy of decision-making, and at the same time reduces the need of potentially harmful and expensive imaging. Most of the existing diagnostic scores have the weakness of being originally based on retrospective data of patients with appendicitis, a small number of patients, or paediatric patients. In retrospective studies, a potential systematic bias involves ignoring the number and outcome of non-operated patients presenting with clinical suspicion of appendicitis. In children, in comparison to adults, the diagnostic limit of leukocyte count and differential diagnosis of acute abdominal pain vary, and depend on age [22,23].

The aim of this study, based on prospectively collected data of adult patients, was to design a new scoring system with easily available variables for more accurate diagnosis of acute appendicitis. The goal was to, on one hand, to recognize patients in need of urgent surgery without delay, and on the other hand, to avoid the unnecessary risks and costs of surgery in non-appendicitis patients. Additionally, the approach aims at avoiding unnecessary ionising radiation. Most importantly, patient’s sex and the time passed since the onset of symptoms to physical examination and obtaining the blood samples is included in the new scoring system. Previous diagnostic scoring systems overlook these important considerations.

## Methods

### Patients

The data were prospectively collected over a period from January 18th 2011 to January 2nd 2012 in a large care facility providing both secondary and tertiary level of surgical care in Finland. During the data collection period 13396 surgical patients visited emergency department. All patients admitted into emergency department for suspected appendicitis or pain in the right lower abdominal quadrant (RLQ) were initially included into the study.

Surgeons with their level of experience varying from first year residents to experienced specialists collected the basic data for the construction of the score during the initial examination at the emergency department.

The collected data included clinical findings (tenderness in RLQ, guarding in RLQ, and body temperature), and symptoms (pain in RLQ, migration of pain, vomiting and anorexia), together with laboratory test results (C-reactive protein (CRP), leukocyte count, proportion of neutrophils), as well as time passed between the onset of symptoms to clinical evaluation. In addition, the surgeons were requested to estimate the probability of appendicitis on clinical basis only with three-step scale: likely, possible, or improbable. Before this estimation, no diagnostic imaging was performed.

The research data consist of 829 data collection forms. Out of these patients 103 lacked neutrophil count, and one lacked CRP count. Results of imaging were evaluated by in-house radiology residents. Details of in-hospital delay, histological findings of the appendix, and final diagnoses were retrieved from a hospital patient data database.

Patients’ medical records were reviewed after a minimum of 2 weeks after the admission to confirm the final diagnosis. If the patient was readmitted during these two weeks, and was diagnosed with acute appendicitis or appendicular abscess, the final diagnosis was classified as acute appendicitis.

The decision to operate was made by the surgeon on duty on the basis of clinical suspicion, after abdominal CT or ultrasound. No scoring systems were used during the data collection period. The optional diagnostic imaging performed as well as the surgical method for appendectomy (open or laparoscopic) was at surgeon’s discretion. The diagnosis of acute appendicitis was based on histological examination showing transmural infiltration of neutrophils in the appendix. At the study hospital, all macroscopically normal appendices are invariably removed for histological examination if no other significant pathology is found during the operation. The operative procedures were therefore in all cases, where appendicitis was preoperatively suspected, registered as appendectomies - not as diagnostic laparoscopies. For the study, complicated appendicitis was defined as perforated appendix or appendicular abscess. Three patients with appendicular abscess were initially treated non-operatively and thus did not have histopathological diagnosis. They were classified as acute appendicitis based on CT findings.

No written informed consent for participation in the study was requested from participants because the study had no influence on the actual diagnostics or treatment of the patients. The data collection and research protocol was approved by the ethics board of Hospital District of Helsinki and Uusimaa.

### Construction of the diagnostic score

The diagnostic score for acute appendicitis was constructed by logistic regression analysis. Continuous laboratory values (CRP, leukocyte count and proportion of neutrophils) were categorized into 4 categories. Cut-off points for the categories were determined by using receiver operating characteristics (ROC) analysis. Cut-off for abnormal body temperature was determined using ROC analysis. Because the distributions of CRP values were significantly different in patients with symptoms less than 24 hours and in patients with symptoms more than 24 hours the cut-off values for CRP were determined in these 2 subsets of patients separately. A multiple imputation for missing neutrophil and CRP data was done. A backward logistic regression analysis included all signs and symptoms, duration of symptoms and categorized laboratory values. Duration of symptoms was used as a variable and an interaction term with categorized CRP values. Being a fertile aged woman (16–49 years old) was included as a variable and an interaction term for all signs and symptoms. Final step of backward stepwise logistic regression with multiple imputed pooled data resulted in statistically significant factors for construction of the score. Points for the score were obtained from regression coefficients by multiplying by 2 and rounding to the nearest integer. Please see Additional file 1 for a table of regression coefficients and the resulting points of the score.

### Evaluation of the score

For comparison Appendicitis Inflammatory Response score (AIR) [16] and Alvarado score [15] were calculated for each patient. The scores were compared by ROC analysis, and the area under ROC curve was determined. The score performance was also evaluated in three clinically different subsets of patients, i.e., in patients with low clinical suspicion, in patients with possible appendicitis and in patients with clinically high suspicion of appendicitis according to surgeons at the emergency department. When evaluating the accuracy of the initial decisions of the surgeons, the use of radiological examinations, final diagnosis and the time taken for decision to operate were considered.

### Statistical analysis

Statistical analysis was made with IBM**®** SPSS**®** Statistics 20.0 for Macintosh (IBM Corporation, Armonk, NY, USA). For the construction of the score backward stepwise logistic regression analysis was used with multiple imputation of missing values. The three different cut-off values in the analysis were determined using ROC analysis. The point where sensitivity and specificity were closest determined the first cut-off value. Other cut-off values included a point with both high sensitivity (over 90%) and at least 30% specificity, and another point with high specificity (over 85%) with at least 20% sensitivity. The cut-off value within these sensitivity and specificity limits was chosen where diagnostic odds ratio (positive likelihood ratio/negative likelihood ratio) was the highest. In case there was no point of high specificity within the sensitivity limit, the section of the ROC-curve with steepest slope determined the cut-off points. Accordingly, in case of no point of high sensitivity within the predefined specificity limit, the section of the ROC curve with gradual raise determined the cut-off points. Patients with missing data were excluded from ROC analysis and from analysis of diagnostic performance. Different scores were compared by ROC-analysis. The diagnostic performance of the new score was compared with initial clinical diagnoses in three clinical subsets of patients using McNemar’s test.

## Results

### Patients

Of the 829 study patients, 393 (47.4%) had appendicitis. Other diagnoses included non-specific abdominal pain for 259 patients, urinary tract infection for 29, acute gastroenteritis for 24, acute diverticulitis for 19, ovarian cyst for 17, gynecological infections for 11, and acute cholecystitis for 9. Other specific diagnoses were found in 68 patients.

Altogether 477 (57.5%) appendectomies were performed out of which 87 (18.2%) were negative. Twenty-nine (3.5%) patients underwent surgery for other indications and 323 (39.0%) patients were managed non-operatively including 3 patients with appendicular abscess (Table 1).

**Table 1:** Patients’ characteristics

	**All**	**Women**	**Men**
**All patients**	829	483	346
Age - median, interquartile range (range)	32, 25–47 (16–97)	31, 24–47 (16–97)	33, 26–47 (16–83)
Operative treatment	506 (61.0%)	272 (56.3%)	234 (67.6%)
**Patients with appendicitis**	392 (47.3)	185 (38.3)	207 (59.8)
Complicated appendicitis	94 (24.0)	50 (27.0)	44 (21.3)
**Non-appendicitis patients**	437 (52.7)	298 (61.7)	139 (40.1)
Operated on for suspected appendicitis	88	67	21
Therapeutic operation*	8 (1.8)	7 (2.3)	1 (0.7)
Operated on for other indications	29 (6.6)	21 (7.0)	8 (5.8)
Non-operative management	320 (73.2)	210 (70.5)	110 (79.1)

*Other diagnosis than acute appendicitis in histopathological examination e.g. tumor of the removed appendix.

### Diagnostic performance of the score

The Adult Appendicitis Score variables and the respective score points are presented in Table 2. The complete score was calculated for 725 patients with full patient data. Of these patients, 343 had appendicitis. Based on the chosen cut-off values in the ROC analysis, patients were classified into three groups corresponding to probability of appendicitis: high (≥16 points), intermediate (11–15 points), and low (0–10 points).

**Table 2:** Adult appendicitis score

**Symptoms and findings**		**Score**
Pain in RLQ		2
Pain relocation		2
RLQ tenderness		3/1*
Guarding	Mild	2
	Moderate or severe	4
**Laboratory tests**		
Blood leukocyte count (×10^9^)	> = 7.2 and <10.9	1
	> = 10.9 and <14.0	2
	> = 14.0	3
Proportion of neutrophils (%)	> = 62 and < 75	2
	> = 75 and < 83	3
	> = 83	4
CRP (mg/l), symptoms < 24 h	> = 4 and <11	2
	> = 11 and <25	3
	> = 25 and <83	5
	> = 83	1
CRP (mg/l), symptoms > 24 h	> = 12 and <53	2
	> = 53 and <152	2
	> = 152	1

RLQ - the right lower abdominal quadrant.

*Men and women age 50+/women, age 16–49.

The score classified 227 patients into the high probability group. This group comprised 199 appendicitis patients and 28 false positives, providing 92.7% specificity in the high probability group. Of all 343 appendicitis patients, 58.0% were classified into the high probability group.

Within the high probability group, patients with 18 points or more represent a subgroup of extra high probability of appendicitis. In this subgroup including 95 (27.7%) of the 343 appendicitis patients, the specificity was 97.6%, with positive likelihood ratio of 11.5 (Table 3).

**Table 3:** (4) Comparison of new score, appendicitis inflammatory response (AIR) score and Alvarado-score in the diagnosis of acute appendicitis

	**Sensitivity (%)**	**Specificity (%)**	**LR+**	**LR-**	**DOR**
**New score**					
**> = 11**	95.9	54.2	2.1	0.076	27.7
**> = 16**	58.0	92.7	7.9	0.45	17.5
**> = 18**	27.7	97.6	11.5	0.74	15.6
**AIR-score**					
**> = 5**	83.1	63.1	2.3	0.27	8.4
**> = 9**	14.6	97.1	5.0	0.88	5.7
**Alvarado-score**					
**> = 4**	98.0	27.7	1.4	0.072	18.8
**> = 7**	68.8	76.4	2.9	0.41	7.1
**> = 9**	27.4	94.2	4.7	0.77	6.1

LR + - positive likelihood ratio.

LR- – negative likelihood ratio.

DOR – diagnostic odds ratio.

277 patients (38.2%) were classified into the group of intermediate probability, and 130 (46.9%) of these patients had appendicitis.

Finally, 221 patients (30.5%) scored low probability of appendicitis. Of them, 14 patients had appendicitis (6.3%), including no cases of complicated appendicitis.

**Figure 1:**
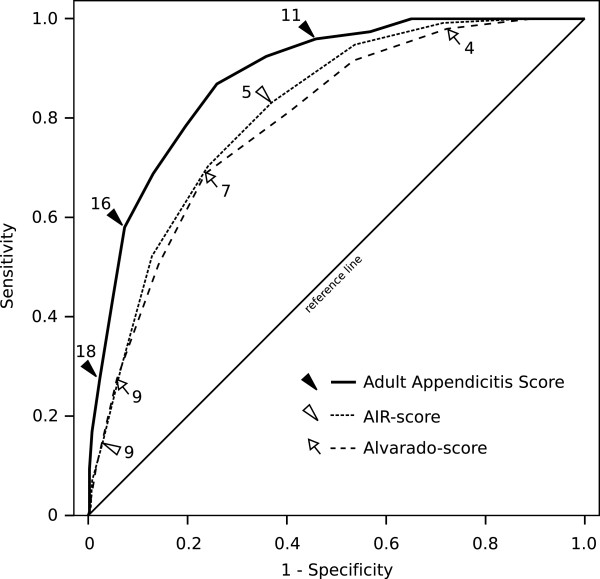
ROC-curves presenting the comparison of the new Adult Appendicitis Score (AUC 0.882 (95% CI 0.858 – 0.906)) compared with Alvarado score (AUC 0.790 (0.758 – 0.823)) and Appendicitis inflammatory response score (AUC 0.810 (0.779 – 0.840)).

The new score was compared to the Alvarado-score and the AIR-score using ROC analysis and area under ROC curve (AUC). In this comparison, the new score had significantly better value of AUC than the others, indicating improved ability of the new score to correctly classify those with and without appendicitis (Figure 1). Using cut-off values of each scoring system chosen by authors in original articles, the diagnostic odds ratio (DOR) was clearly best for the new score (Table 3).

### Clinical diagnostics

In the score population (n = 725), based on the initial physical examination in the emergency care unit, the on-duty surgeons considered appendicitis to be likely in 241, possible in 355, and improbable in 129 patients. Of those 241 patients considered likely appendicitis, 188 (78.0%) had appendicitis. Of the 355 considered possible appendicitis patients 145 (40.8%), and of the 129 considered improbable appendicitis patients, 10 (7.8%) turned out to have appendicitis. Among 241 patients considered likely appendicitis, 142 (58.9%) patients had score 16 or higher. Out of these patients 133 (93.7%) had appendicitis. Whereas in 99 patients with clinically considered likely appendicitis but score below 16, only 55 (55.6%) patients had appendicitis. Eighty-one (22.8%) out of 355 patients with clinically considered possible appendicitis had score 16 or higher. Sixty-three (77.8%) out of these had appendicitis.

A total of 421 patients were operated on for suspected appendicitis, comprising of 207 patients in the high probability category according to the new score, 176 in the intermediate probability group, and 38 in the low probability group. Of the appendectomies in the high probability group, 4.3% (9 of 207), in the intermediate probability group 27.2% (48 of 176), and in low probability group 63.1% (24 of 38) were negative.

The performance of the new score was compared to the performance of diagnostics after the initial physical examination only, within 6 hours of the initial physical examination, and within 24 hours of the initial examination. In all three comparisons, scoring 16 or higher with the new score had significantly better specificity (Table 4).

**Table 4:** Comparison of the new score and clinical diagnostics

**Assessment of appendicitis**	**Sensitivity (%)**	**Specificity (%)**	p-value for sensitivity*	p-value for specificity**
**At clinical examination**^**†**^	54.8	86.1	0.363	0.002
**At 6 h from clinical examination**^**‡**^	55.4	86.6	0.529	0.007
**Within the first 24 hours**	98.5	78.4	<0.001	<0.001
**Score > =16**	58.0	92.7		

*Comparison with sensitivity of the new score > =16 (McNemar test).

**Comparison with specificity of the new score > =16 (McNemar test).

^†^High probability of appendicitis based on clinical examination alone.

^‡^Clinical diagnosis of appendicitis within 6 h from clinical examination.

### CT

In the score population (n = 725), altogether 152 patients (20.9%) underwent CT. Based on the new score, 48 of them were in the high probability group, 69 in the intermediate probability group, and 35 in the low probability group. In-house radiology residents found 69 cases of appendicitis. Of all CT-scans performed, 6 resulted in false positives, and 3 false negatives, for appendicitis.

Sensitivity of CT was in the score population 95.5%, and specificity 93.0%.

## Discussion

The new Adult Appendicitis Score classified half of the actual appendicitis patients in high probability group, with specificity comparable to CT, and better than actual diagnostics at the emergency department. Nearly one third of patients with appendicitis were classified as in the extra high probability group. Specificity in this group was superior to specificity of CT in our study population, and superior or comparable to specificity of CT overall [24]. Only few patients with appendicitis, among them no complicated cases, were classified into the low probability group.

The new score was in our study population superior to previously published Alvarado Score, AIR-score, and decisions surgeons made based on physical examination and diagnostic imaging. The difference to other scoring systems and the resulting improved diagnostic performance is based on well-known features of appendicitis; our score is the first to take into account the differences in diagnostics between sexes, and also the first to take into account the time passed between the onset of symptoms and taking the laboratory samples. In addition, strength of the new score is its being based on prospectively collected data of all patients with RLQ-pain, not only those operated on for suspected appendicitis.

Compared to the new score, AIR-Score classified only a small minority of patients (8.4%) into high probability category, and therefore the need of diagnostic imaging, consultations, and further observation (or the rate of negative appendectomies) remains the same as without scoring. Alvarado-score classifies nearly half of all patients into the high probability category. In this category, however, diagnostic accuracy of the Alvarado-score reaches neither the accuracy of overall clinical diagnostics nor the accuracy of the new score. In the original publication of the AIR-score, the AIR-score performed better than in our study population. The reason for this could be in different age distribution or otherwise different inclusion criteria of patients.

Notably, the decisions to operate based on physical examination only result in a high rate of negative appendectomies [3-5]. A negative appendectomy can lead to severe morbidity and even mortality. Even without complications it is associated with unnecessary disability and costs.

The increasing use of CT has helped to reduce the amount of negative appendectomies. Not all emergency care units have 24-hour availability of CT, whereas in some units, nearly all patients suspected of appendicitis undergo CT [4]. The results of this study indicate, that many of these CT-scans, as well as referrals to units with 24-hour CT, could be avoided using the new scoring system.

In the study hospital CT, ultrasound, and magnetic resonance imaging are available at all times, but are traditionally not frequently used in diagnostics of acute appendicitis.

With the new score, the majority of the patients were classified in either the high or the low probability group, and the clear separation of likelihood reduces the need for diagnostic imaging significantly. CT and ultrasound are known to have least benefit for patients with highest and lowest probability of appendicitis [24]. Furthermore, the risks of radiation should be carefully considered in clinical practice; appendicitis is especially common in young people, where accumulated ionizing radiation doses provide greatest total risk over lifetime.

In a comprehensive review on radiation doses and involved cancer risk, gender-averaged percentage lifetime radiation-attributable cancer risk from a single abdominal CT-scan was estimated 0.06% for a 25 year old patient. Clinical practice guidelines and selective imaging strategies for diagnosis of paediatric appendicitis were used in this review as an example of possible means to reduce CT usage [14].

Ultrasound is a safe, radiation-free method, but gives inferior differentiation of appendicitis compared to CT. In a review of CT versus graded compression US in the diagnostics of acute appendicitis the mean respective sensitivities of CT and ultrasound were 91% and 78%, and the respective specificities 90% and 83% [24].

Results of our study are promising; the new score can easily be adopted for clinical practice, providing a significant speed up, and reduction of morbidity as well as cost. All the variables are easily available for the score, and the counting itself can be performed with a computer or a regular calculator. To make scoring even easier, it would be simple to develop a web-based application suitable for counting the score.

Patients in the subgroup of extra high probability (score ≥18) could be scheduled to surgery without any further investigations or follow-up. In high probability group (score ≥16), diagnostic accuracy improved when clinical assessment was taken into account. Accuracy was best when score results and the actual diagnostics were combined. When both the score and the surgeon suggested surgery, half of all appendicitis patients were recognised with rate of negative appendectomies as low as 4.3%.

In the group of intermediate probability for appendicitis (11–15 points), the study shows that all patients would need further examinations. This is the group of patients that benefits most from imaging, in-hospital follow-up and gynaecological consultations; nearly half of them have appendicitis, and many of them are in need of treatment for other reasons.

Patients in the low probability group may need further observation, imaging, or consultations for some other reasons than suspected appendicitis. They may also have appendicitis, but this is not a frequent cause for their discomfort. If after physical examination and laboratory tests no serious concerns exist, these patients can be discharged.

Of the 829 patient data sets collected for this study, 103 lacked neutrophil counts and one lacked CRP. Neutrophil count was not routinely checked for every patient with abdominal pain in the care unit where this study was performed and thus was easily omitted by the surgeons. Some patients with RLQ pain were not included in the study for non-compliance of a minority of the surgeons, i.e., no returned data forms. This bias is not systematic because, e.g., time of admission did not affect inclusion of patients, but instead depended on the appointed surgeon.

Scoring for diagnosing appendicitis is fast and easily available to all physicians involved in treatment of abdominal pain patients. Some limitations can, however, weaken the accuracy of the new score; patients may have difficulty in defining the time of onset of symptoms, and physician’s report on the severity of guarding can be uncertain with inexperienced providers.

The study was performed in a single surgical center, and only adult patients were included. Validation of the new score in another prospective patient material, especially in another surgical unit, would make the score more reliable for clinical use.

## Conclusions

The new Adult Appendicitis Score presented here differs from previous scoring systems by taking into account the important effects of gender and duration of symptoms. It helps to categorize patients accurately into three different groups. In addition to the majority of patients that can be safely discharged from the emergency department or assigned directly to surgery, it identifies a group (38% of all patients) that would benefit from further diagnostic studies such as CT.

## Competing interests

The authors declare that they have no competing interests.

## Authors’ contributions

All three authors contributed to the design of the study, and read and approved the final version of the manuscript. HS collected the data and participated to data analysis, drafted the manuscript, and submitted the final version of the manuscript. PM analyzed the data together with HS, and drafted the manuscript. AL helped to draft the manuscript, and revised the manuscript critically.

## Pre-publication history

The pre-publication history for this paper can be accessed here:

http://www.biomedcentral.com/1471-230X/14/114/prepub

## Supplementary Material

Additional file 1 Construction of adult appendicitis score. Click here for file
