# Supplementary material for: A new adult appendicitis score improves diagnostic accuracy of acute appendicitis - a prospective study
Source: BMC Gastroenterol. 2014 Jun 26;14:114. doi: 10.1186/1471-230X-14-114 (PMC4087125; doi:10.1186/1471-230X-14-114)
Supplement: Additional file 1 — Construction of adult appendicitis score. [file 1471-230X-14-114-S1.docx]

**Additional file 1: Construction of Adult Appendicitis Score**

| **Symptoms and findings** |  | **Regression coefficient** | **p-value** | **Score** |
| --- | --- | --- | --- | --- |
| Pain in RLQ |  | 1.249 | <0.001 | 2 |
| Pain relocation |  | 1.068 | <0.001 | 2 |
| RLQ tenderness |  | 1.667 | 0.045 | 3 |
| RLQ tenderness * women, age 16-49 |  | -1.312 | <0.001 | 1^†^ |
| Guarding | none | reference |  |  |
|  | mild | 1.115 | 0.001 | 2 |
|  | Moderate or severe | 1.768 | 0.001 | 4 |
| **Laboratory tests** |  |  |  |  |
| Blood leukocyte count (x10^9^) | <7.2 | reference |  |  |
|  | >=7.2 and <10.9 | 0.312 | 0.348 | 1 |
|  | >=10.9 and <14.0 | 0.822 | 0.021 | 2 |
|  | >=14.0 | 1.365 | <0.001 | 3 |
| Proportion of neutrophils (%) | <62 | reference |  |  |
|  | >=62 and < 75 | 1.143 | 0.001 | 2 |
|  | >=75 and < 83 | 1.368 | <0.001 | 3 |
|  | >=83 | 2.062 | <0.001 | 4 |
| CRP (mg/l), symptoms < 24h | <4 | reference |  |  |
|  | >=4 and <11 | 1.052 | 0.009 | 2 |
|  | >=11and <25 | 1.626 | <0.001 | 3 |
|  | >=25 and <83 | 2.533 | <0.001 | 5 |
|  | >=83 | 0.385 | 0.456 | 1 |
| CRP (mg/l), symptoms > 24h | <12 | reference |  |  |
|  | >=12 and <53 | 1.228 | <0.001 | 2 |
|  | >=53 and <152 | 1.202 | <0.001 | 2 |
|  | >=152 | 0.748 | 0.074 | 1 |

RLQ - the right lower abdominal quadrant

^†^Score for RLQ tenderness for women, age 16-49 is based on the sum of regression coefficients of RLQ tenderness (1.667) and RLQ tenderness * women, age 16-49 (-1.312).
